# An unusual variant of the abducens nerve duplication with two nerve trunks merging within the orbit: a case report with comments on developmental background

**DOI:** 10.1007/s00276-015-1573-x

**Published:** 2015-10-26

**Authors:** Grzegorz Wysiadecki, Michał Polguj, Mirosław Topol

**Affiliations:** Department of Normal and Clinical Anatomy, Interfaculty Chair of Anatomy and Histology, Medical University of Lodz, ul. Narutowicza 60, 90–136 Łódź, Poland; Department of Angiology, Interfaculty Chair of Anatomy and Histology, Medical University of Lodz, ul. Narutowicza 60, 90-136 Łódź, Poland

**Keywords:** Abducens nerve, Variations, Cranial nerve development, Lateral rectus innervation pattern

## Abstract

This study reports the first case of abducens nerve duplication along its entire intracranial course, ending within the orbit. A distinct abducens nerve duplication reaching the common tendinous ring (annulus of Zinn), as well as another split within the intraconal segment of the nerve have been revealed. Additionally, two groups (superior and inferior) of abducens nerve sub-branches to the lateral rectus muscle were visualised using Sihler’s stain. The analysed anatomical variation has never been reported before and it seems to be in the middle of the spectrum between the cases of duplication occurring only within the intracranial segments of the abducens nerve found in the literature and those continuing throughout the whole course of the nerve. Abducens nerve duplication may be treated as a relic of early stages of ontogenesis. Such a variant might result from alternative developmental pathways in which axons of the abducens nerve, specific for a given segment of the lateral rectus muscle, run separately at some stage, instead of forming a single stem.

## Introduction

The abducens nerve (CN VI) typically occurs as a single trunk. However, multiple exceptions to this anatomical norm have been described in medical literature, including absence of CN VI [1], split of CN VI into branches in the cavernous sinus [2, 3], as well as different variants of duplication [2, 4–8]. Due to the complex anatomical relationships of CN VI, three intracranial segments (cisternal or subarachnoid, gulfar or petroclival and cavernous) and two orbital segments (fissural and intraconal) have been distinguished on its course [1, 4].

The frequency of CN VI duplication reported by different authors ranges from 5 to 28.6 % [5]. Kshettry et al. analysing the literature data, estimated the average incidence of CN VI duplication to be 7.6 % (35 out of 462 analysed cases) [7]. Although a number of variants of CN VI duplication have been described, in almost all cases both trunks (some authors use the term ‘roots’ [5, 7] or ‘branches’ [8]) united to form a single stem within one of the intracranial segments of the nerve—most frequently in the cavernous sinus [2, 4–8].

To date, only one case of CN VI duplication starting at the pontomedullary sulcus and extending beyond the intracranial segments has been reported in literature (Jain’s series from 1964) [6]. In the case at hand, there were two trunks which travelled separately up to the lateral rectus muscle along the entire course of CN VI [6]. Thus, occurrence of CN VI duplication along its entire intracranial course extending as far as to the intraorbital segments of the nerve is extremely rare.

The presented study is the first case report of CN VI duplication along the entire intracranial course, ending within the orbit (at the level of the fissural segment of the nerve) with another slight split in the intraconal segment. The importance of the presented variation, apart from clinical implications resulting from the occurrence of an unexpected second branch, which may be injured during surgical procedures, is primarily cognitive. Bergman et al. emphasised that anatomical variations ‘teach us about our development, and something about our genetic heritage’ [9].

The aim of the study was to observe in detail the course and topographic relations of both trunks of a duplicated CN VI, as well as the distribution of sub-branches reaching the lateral rectus muscle. The possible developmental background of the observed variation was also analysed.

## Case description

A 68-year-old male cadaver was subjected to routine dissection for scientific and teaching purposes. No head injury or surgical interventions were detected upon a visual inspection of the body. After eyelid elevation symmetrical placement of the eyeballs was seen on both sides. The skull was opened with extreme caution in order to preserve the cranial nerves intact, using a protocol described by Long et al. [10]. Upon exposure of the posterior cranial fossa, duplication of CN VI was revealed on the right side, with two nerve trunks of similar diameter (Table 1) emerging directly from the brain stem. Both trunks travelled in the subarachnoid space separately. One of these trunks was located more laterally and pierced the clival dura mater superior to the other one located more medially. The distance between the dural entry points of both trunks was 0.68 mm.

**Table 1:** Summary of the results of measurements performed for both trunks of the duplicated abducens nerve

Measured feature (mm)	DE-PCP	DE-TG	DE-IAO	Diameter (within subarachnoid space)
Lateral trunk	19.2	4.3	19.3	0.72
Medial trunk	19.7	6.8	20.5	0.84

*DE*-*PCP* distance between the dural entry point of an individual trunk of the duplicated abducens nerve and the apex of the posterior clinoid process, *DE*-*TG* distance between the dural entry point of an individual trunk of the duplicated abducens nerve and the inferior border of the trigeminal porus (the trigeminal nerve entrance to Meckel’s cave), *DE*-*IAO* distance between the dural entrance of an individual trunk of the duplicated abducens nerve and the central part of the internal acoustic opening

At this stage of the dissection, the distances between the dural entry points of the duplicated CN VI and the selected topographical landmarks were measured. The following reference points were used: the apex of the posterior clinoid process, the inferior border of the trigeminal porus (the trigeminal nerve entrance to Meckel’s cave) and the central part of the internal acoustic opening (Table 1). The measurements were taken using Digimatic digital caliper (Mitutoyo Company, Kawasaki-shi, Kanagawa, Japan).

The next stage of the dissection was performed at 2.5× magnification obtained with HEINE^®^ HR 2.5× High Resolution Binocular Loupe (HEINE Optotechnik GmbH & Co. KG, Herrsching, Germany). The clival dura mater was carefully dissected to expose the petrosphenoidal ligament of Grüber and the course of CN VI within the petroclival venous confluence. After reaching the petroclival venous confluence, the medial trunk of the duplicated CN VI ran below the petrosphenoidal ligament (Grüber’s ligament), within Dorello’s canal, whereas the other trunk, located laterally, ran above this ligament.

**Fig. 1:**
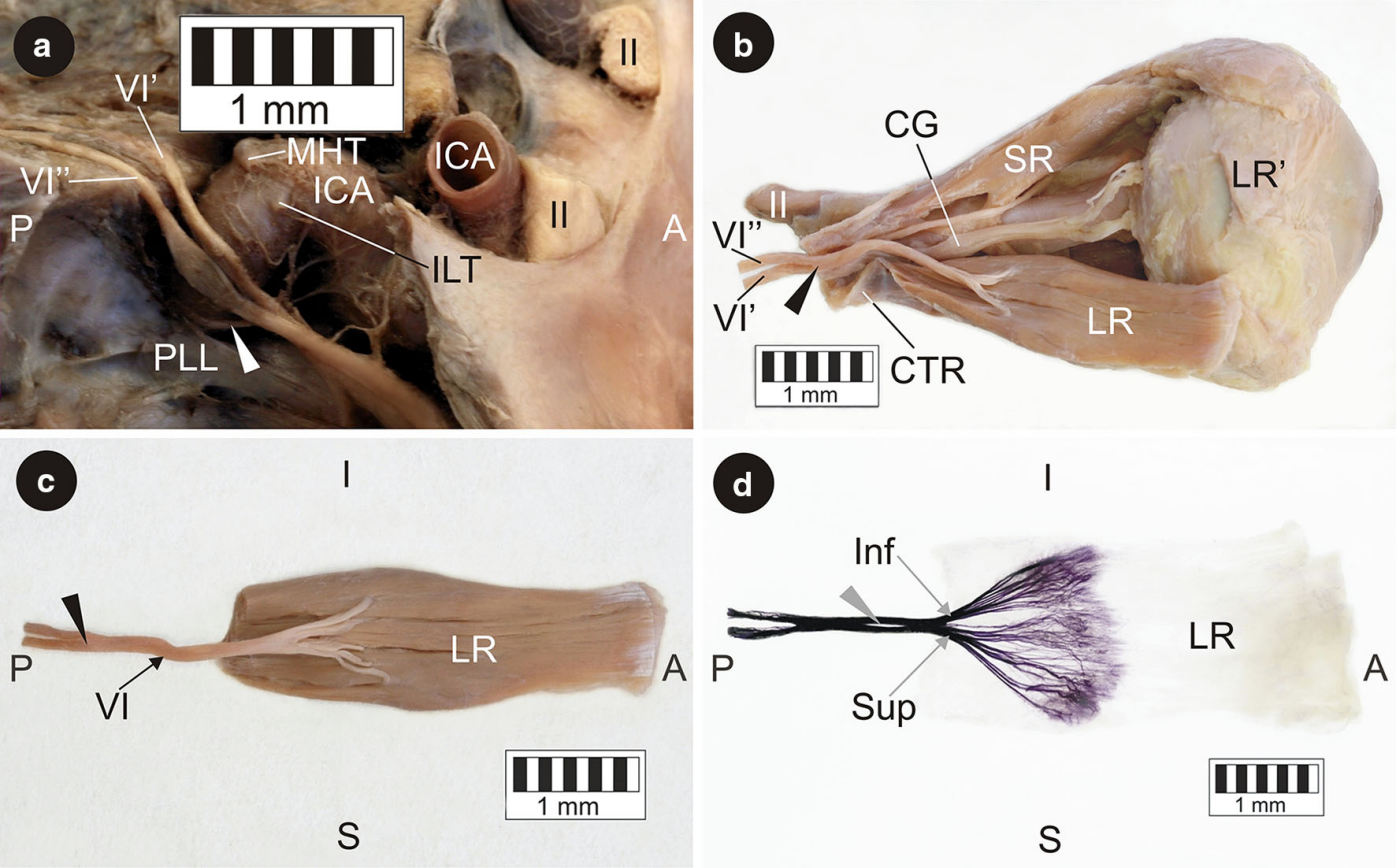
The course and innervation pattern of the duplicated abducens nerve. **a** Intracranial segments. The clival dura mater and the lateral wall of the cavernous sinus were removed. The main branches of the cavernous segment of the internal carotid artery were carefully removed during the dissection in order to better visualise the course of the abducens nerve. *White arrowhead* internal carotid plexus. **b** Intraorbital segments. A lateral incision of the common tendinous ring exposed the point where both trunks of the duplicated abducens nerve merged. *Black arrowhead* the site of fusion of both trunks of the duplicated abducens nerve. **c** Isolated lateral rectus muscle specimen. The inner surface of the muscle was visualised along with the abducens nerve sub-branches reaching it. *Black arrowhead* the site of fusion of both trunks of the duplicated abducens nerve. **d** Intramuscular innervation pattern of the lateral rectus muscle. Sihler’s staining. View of the internal muscle surface. A slight deformation of the muscle results from the technological process of staining. *Grey arrowhead* short ‘split’ within intraconal segment of the abducens nerve. *A* anterior, *P* posterior, *I* inferior, *S* superior, *II* optic nerve, *VI* single trunk (intraconal segment) of the abducens nerve, *VI’* medial trunk of the duplicated abducens nerve, *VI”* lateral trunk of the duplicated abducens nerve, *CG* ciliary ganglion, *CTR* common tendinous ring, *ICA* internal carotid artery, *ILT* origin of the inferolateral trunk, *LR* lateral rectus muscle, *LR’* insertion of the lateral rectus, *MHT* origin of the meningohypophyseal trunk, *PLL* petrolingual ligament*, SR* superior rectus muscle, *Inf* sub-branches to the inferior compartment of the lateral rectus, *Sup* sub-branches to the superior compartment of the lateral rectus

Both nerve trunks entered the cavernous sinus and adhered to the ascending portion of the cavernous segment of the internal carotid artery (Fig. 1a). At this level both trunks received communicating branches from the internal carotid plexus and crossed each other in the further course (Fig. 1a). Finally, both trunks of the duplicated CN VI passed through the superior orbital fissure and united to form a single stem within the common tendinous ring (Fig. 1b). The merging point of both CN VI trunks was located most laterally in relation to other structures found within the annulus of Zinn (Fig. 1b). Thus, both trunks united only after entering the orbit within the fissural segment of the nerve. The mean diameter of CN VI within the intraconal segment was 1.38 mm. An additional slight split of 3.86 mm was observed in the intraconal segment of CN VI, which merged into a single nerve supplying only the lateral rectus muscle (Fig. 1c).

To visualise the intramuscular distribution of CN VI sub-branches, the isolated lateral rectus muscle was stained using Sihler’s whole mount nerve staining technique according to the procedure described by Mu and Sanders [11]. Taking into account the small muscle mass, the time of individual staining stages was modified (destaining—4 weeks, decalcification—2 weeks, staining—2 weeks). Thus visualised sub-branches running to the lateral rectus demonstrated a division into two groups, supplying superior and inferior compartments of the muscle, respectively (Fig. 1d). Both groups of sub-branches formed a characteristic ‘tufty’ branching (arborisation) pattern within the proximal half of the lateral rectus muscle (Fig. 1d). At the same time, the application of Sihler’s staining allowed better visualisation of the splitting within the intraconal segment of CN VI (Fig. 1d).

## Discussion

Knowledge of CN VI anatomical variations may help reduce the risk of iatrogenic injury to this nerve. All the more so as progress in modern imaging techniques allows for a precise evaluation of anatomical structures. For instance, Li et al. documented the usefulness of 3D-SPACE sequence MR scanning in evaluation of individual CN VI segments [12]. Moreover, a shift in the location of a duplicated CN VI in relation to selected topographical landmarks may prove to be of utmost importance during neurosurgical procedures. Such a variation may be associated with a decreased distance to the trigeminal nerve entrance to Meckel’s cave (trigeminal porus) in comparison with a typical course of the CN VI, as well as close proximity to a posterior clinoid process [2].

**Fig. 2:**
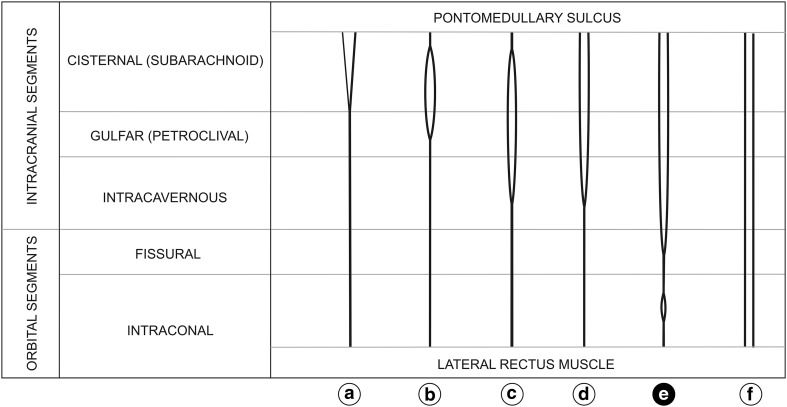
Variants of abducens nerve duplication along with a schematic illustration of its course within individual nerve segments

Figure 2 demonstrates different variants of CN VI duplication described in the literature. Both parts of a duplicated CN VI may leave the brainstem separately (Fig. 2, variants a, d, e and f) or as a single nerve splitting into two divisions in the subarachnoid space (Fig. 2, variants b and c) [2, 4–6, 8]. Occasionally, both trunks of a duplicated CN VI may pass through the same dural entry point (Fig. 2, variant a) [1], but usually they pierce the clival dura mater separately (Fig. 2, variants b–f) [2, 4–8]. Within the petroclival venous confluence one of the trunks of a duplicated CN VI may run outside of Dorello’s canal, above the petrosphenoidal ligament [2, 4–6, 8]. In a vast majority of cases described in the literature, both trunks of a duplicated CN VI united in the cavernous sinus (within the intracavernous segment of the nerve—Fig. 2, variants c and d) [2, 6–8]. However, there are reported cases of both trunks merging in Dorello’s canal (within the gulfar segment of the nerve—Fig. 2, variant b) [2]. Along the course of a duplicated CN VI both trunks may be of a similar diameter or one of them may be thinner [2, 5, 8]. CN VI duplication on its entire course described by Jain (Fig. 2, variant f) [6], as well as the case of CN VI duplication with both trunks merging within the common tendinous ring described in this report (Fig. 2, variant e) are unusual anatomical variations.

All variants of CN VI duplication may be accounted for by mechanisms controlling the development of cranial nerves. One of key stages during the development of somatic motor cranial nerves is correct establishment of cranial nerve motor nuclei [13]. Further on, growing nerve fibres must find their appropriate targets, which depends on undisturbed axonal growth and guidance (pathfinding) from the motor nuclei to developing muscles [13–15]. Normal growth of the nerve from its formation to the mature structure is regulated by a large number of molecular and cellular mechanisms [13–15]. Defects in cranial motor neuron development and axon navigation may be the cause of abnormalities in the wiring of extraocular muscles, resulting in eye movements deficits [13, 15]. However, there is evidence that CN VI duplication is an anatomical variant which falls within the norm. This view is supported by a report by Kim and Hwang [16], who described a completely normal eye movement in a patient with unilateral duplication of CN VI observed by MRI.

Demer et al. [14], Guthrie [15], Peng et al. [17] and da Silva Costa et al. [18] indicated that the lateral rectus muscle may be composed of functionally distinct superior and inferior compartments (zones) which can be independently controlled by the nervous system and selectively activated. The assumption that each of the two compartments of the lateral rectus muscle is a separate target for migrating axons of the developing CN VI would cast new light on the cases of duplication of the nerve. During development, the lateral rectus muscle is formed from two individual myotomes and in adults it usually has dual headed origin [17]. At early stages of the development, hindbrain (rhombencephalon) demonstrates segmental structure. Its individual segments are called rhombomeres. CN VI is formed by motor neurons derived from progenitor cells of rhombomeres r5 and r6 [15]. As shown in previous studies, neuronal precursors differentiating within individual rhombomeres demonstrate strictly defined specificity (determined by a precise target organ) [1, 15]. It is likely that axons migrating to different compartments of the lateral rectus muscle may be characterised by such specificity. Therefore, each compartment of the lateral rectus muscle might interact with a strictly defined group of axons of the developing CN VI. This assumption is supported by Peng’s et al. claim that primary bifurcation of CN VI into superior and inferior groups of sub-branches is ‘external to the lateral rectus on its global surface in the posterior orbit, or even more proximally’ [17]. Peng’s et al. observations are consistent with ours: two groups of sub-branches to the lateral rectus emerged even before entering the muscle (Fig. 1c and d), which was particularly well visualised in this study using Sihler’s stain. Da Silva Costa et al. also speculated that ‘superior and inferior LR zones might be segregated at the motor nucleus and motor nerve levels’ [18]. Hence, CN VI duplication might result from alternative developmental pathways in which this nerve’s axons, specific for a given segment of the lateral rectus muscle, run separately at some stage, instead of forming a single nerve. Such a duplication supports Peng’s et al. hypothesis [17] that ‘CN6 may contain topographically distinct branches that may innervate separate LR functional compartments’.
